# Down-Regulation of Phosphoenolpyruvate Carboxylase Kinase in Grapevine Cell Cultures and Leaves Is Linked to Enhanced Resveratrol Biosynthesis

**DOI:** 10.3390/biom11111641

**Published:** 2021-11-05

**Authors:** Elías Hurtado-Gaitán, Susana Sellés-Marchart, James Hartwell, Maria José Martínez-Esteso, Roque Bru-Martínez

**Affiliations:** 1 Plant Proteomics and Functional Genomics Group, Agrochemistry and Biochemistry Department, Faculty of Science, University of Alicante, 03690 Alicante, Spain; ehurtadog7@gmail.com (E.H.-G.); susana.selles@ua.es (S.S.-M.); mjose.martinez@gcloud.ua.es (M.J.M.-E.); 2 Department of Functional and Comparative Genomics, Institute of Integrative Biology, University of Liverpool, Liverpool L69 7ZB, UK; James.Hartwell@liverpool.ac.uk; 3 Instituto de Investigación Sanitaria y Biomédica de Alicante ISABIAL-Fundación para el Fomento de la Investigación Sanitaria y Biomédica de la Comunitat Valenciana FISABIO, 03010 Alicante, Spain

**Keywords:** PEP carboxylase kinase, resveratrol biosynthesis regulation, elicitation, cell culture, *Vitis vinifera*, Gamay

## Abstract

In grapevine, trans-Resveratrol (tR) is produced as a defence mechanism against stress or infection. tR is also considered to be important for human health, which increases its interest to the scientific community. Transcriptomic analysis in grapevine cell cultures treated with the defence response elicitor methyl-β-cyclodextrin (CD) revealed that both copies of PHOSPHOENOLPYRUVATE CARBOXYLASE KINASE (*PPCK*) were down-regulated significantly. A role for PPCK in the defence response pathway has not been proposed previously. We therefore analysed the control of *PPCK* transcript levels in grapevine cell cultures and leaves elicited with CD. Moreover, phosphoenolpyruvate carboxylase (*PPC*), stilbene synthase (*STS*), and the transcription factors *MYB14* and *WRKY24,* which are involved in the activation of *STS* transcription, were also analysed by RT-qPCR. The results revealed that under CD elicitation conditions *PPCK* down-regulation, increased stilbene production and loss of PPC activity occurs in both tissues. Moreover, *STS* transcripts were co-induced with *MYB14* and *WRKY24* in cell cultures and leaves. These genes have not previously been reported to respond to CD in grape leaves. Our findings thus support the hypothesis that PPCK is involved in diverting metabolism towards stilbene biosynthesis, both for in vitro cell culture and whole leaves. We thus provide new evidence for PEP being redirected between primary and secondary metabolism to support tR production and the stress response.

## 1. Introduction

Grapevine, *Vitis vinifera* L., induces biosynthesis of the phytoalexin stilbene as part of its stress defence response. The most studied stilbene in this response is trans-Resveratrol (trans-3,5,4’-trihydroxystilbene, tR). Stilbenes play important roles in plant responses to both abiotic [1,2] and biotic stresses, since they display strong antimicrobial and antifungal activities [3,4,5]. Many studies have also reported human health benefits resulting from the dietary consumption of stilbenes. tR has been largely studied for its antioxidant, anti-inflammatory, immunomodulatory and anti-angiogenic effects, and for its potential roles in chemoprevention of cancer and cardiovascular diseases [6,7,8].

Stilbene biosynthesis from a sugar carbon source in primary metabolism involves the transformation of sugar into erythrose-4 phosphate (E4P) via the pentose phosphate pathway and phosphoenolpyruvate (PEP) via glycolysis. E4P and PEP are converted to phenylalanine via the shikimate pathway, and phenylalanine then enters the phenylpropanoid pathway to yield 4-coumaryl-CoA, which is the common precursor of phenolic compounds in plants. t-R is synthesized as an end product of the phenylpropanoid pathway by condensation of one molecule of 4-coumaroyl-CoA with three molecules of malonyl-CoA in a reaction catalysed by stilbene synthase (STS) [9].

Stilbene biosynthesis is promoted by a wide range of biotic and abiotic elicitors, which induce transcriptome- and proteome-wide changes involving the enzymes of the Shikimate-phenylpropanoid-stilbenoid pathways, thus enhancing tR accumulation [10,11,12,13]. In addition to these previously studied responses, the whole transcriptome analysis of the response of a grape berry cell culture system to the stress-response elicitors methyl-β-cyclodextrin (CD) alone, or CD combined with methyl jasmonate (CDMJ), identified a differential expression of genes whose potential role in this response had not been reported previously [13]. In this context, transcripts for both copies of phosphoenolpyruvate carboxylase kinase (*VvPPCK1* and *VvPPCK2*) in the grapevine genome, were down-regulated up to 12-fold in response to CD treatment [13].

PPCK is a specific protein kinase that phosphorylates phosphoenolpyruvate carboxylase (PEPC or PPC) on an invariant N-terminal serine residue that is highly-conserved across all plant-type PPCs [14]. PPC itself catalyses the conversion of HCO_3_^−^ plus PEP to OAA and Pi. The OAA is rapidly converted to malate-by-malate dehydrogenase. Phosphorylated PPC is less sensitive to inhibition by L-malate, thereby protecting PPC from feedback inhibition by the ultimate product of its activity [15]. PPC phosphorylation, and subsequently the activity of PPCK, were first identified in plants that perform the metabolic adaptations of photosynthetic CO_2_ fixation known as Crassulacean acid metabolism (CAM) and C_4_ [15,16,17,18]. In both CAM and C_4_ plants, PPC functions as the primary carboxylase for CO_2_ fixation during photosynthesis. In CAM species, PPCK is transcribed and translated de novo each night under the temporal control of the central circadian clock [18,19,20]. Nocturnal phosphorylation of PPC prolongs and sustains high PPC activity throughout the dark period, supporting atmospheric CO_2_ fixation, and resulting in high concentrations of vacuolar malic acid by dawn [21]. In contrast, PPCK in C_4_ plants is induced by light, leading to the phosphorylation of PPC in the leaf mesophyll cells where PPC catalyses primary CO_2_ fixation [22,23,24].

PPC also plays a range of roles in both photosynthetic and non-photosynthetic tissues of all plants. For example, PPC functions in the anaplerotic replenishment of C_4_-dicarboxylic acids used for both energy and biosynthetic metabolism [25,26], and in the response to several types of abiotic stresses, including drought, salt stress and Pi starvation, each of which promote an increase of PPCK and PPC activities [27,28,29]. However, in the context of the response of plant cells to elicitation leading to stilbene biosynthesis in grapevine, a role for PPC and its regulatory protein kinase PPCK has not been reported previously, even though these proteins might represent a key regulatory point at the crossroads between primary and secondary metabolism. Interestingly, in a grape berry cell culture system, early signalling events mediated via a phosphorylation/dephosphorylation cascade induced by both CD and MJ treatment have been shown to be crucial for the induction of stilbene production, showing a dramatic reduction in tR production in grapevine cell cultures under elicitation when the cultures were treated with MAP kinase inhibitors [30].

While broad current knowledge of catalytic enzymes belonging to the shikimate-phenylpropanoid-stilbenoid pathways has facilitated metabolic engineering approaches for enhancing and diversifying stilbene biosynthesis [31,32], relatively few studies have examined the regulation of stilbene biosynthesis. Regulation represents an important alternative strategy for the manipulation of stilbene metabolism. In this context, in grapevine, the MYB and WRKY superfamily of transcription factors (TFs) have been studied due to their ability to bind to and activate the promoters of stilbene synthase (*VvSTS*) genes. *VvMYB14* and *VvMYB15* were reported to activate the *VvSTS29* and *VvSTS45* promoters in a dual luciferase reporter system [33]. Recently, Vanozzi and co-workers (2018) also discovered a member of the WRKY TF family (*VvWRKY24*) that is able to activate the *VvSTS29* promoter in the absence of *VvMYB14* or *VvMYB15* [34]. However, current understanding of the regulation of stilbene biosynthesis represents only the tip of the iceberg, and it remains clear that further studies to decipher the complex regulatory network underpinning the induction of stilbene biosynthesis will open up new possibilities to enhance stilbene production.

The well-known biological roles of PPC and its phospho-regulator PPCK in primary metabolism may be important on the pathway to stilbene biosynthesis, perhaps as part of the very earliest steps of the defence response. For example, the down-regulation of *PPCK* in response to elicitation might effectively reduce the in vivo activity of PPC due to its dephosphorylation by a constitutively active protein phosphatase type 2A (PP2A) [35]. Dephospho-PPC is more sensitive to feedback inhibition by L-malate, and PPC activity would therefore be reduced in vivo, leading to an accumulation of its substrate, PEP. Since PEP is a principal substrate for the shikimate pathway, and the shikimate pathway provides chorismate for phenylalanine production, which in turn supports subsequent phenylpropanoid biosynthesis, the down-regulation of *PPCK* may promote the redirection of PEP towards the shikimate pathway. This proposed biological role of PPCK would increase the carbon flow out of primary metabolism towards secondary metabolic pathways in response to stress. We therefore hypothesize that PPCK is a key regulator of stilbene biosynthesis in response to elicitors, modulating the carbon partitioning between primary and secondary metabolism, where the destination of incoming carbon must be balanced between core cellular requirements and the dynamic response of the plant to changing environmental conditions.

To investigate this potential regulatory role of PPCK in the control of stilbene biosynthesis, we used CD to elicit the defence response and stilbene biosynthesis in *V. vinifera* cv. Gamay cell cultures and leaves. We measured the response to CD elicitation for the transcript abundance of *VvPPCKs* and *VvPPCs*, along with total extractable PPC activity, and also studied whether the known regulatory TFs *VvMYB14* and *VvWRKY24* were co-regulated with *VvSTS* induction and increased stilbene accumulation.

## 2. Materials and Methods

### 2.1. Plant Material

*Vitis vinifera* L. cv. Gamay calli were supplied by Drs. J. C. Pech and A. Latché (ENSA, Toulouse, France) in 1989. The cell line was maintained in both solid and liquid cultures in Gamborg B5 medium as described elsewhere [4].

*V. vinifera* L. cv. Gamay plants were purchased from Victoriana Nursery Gardens (Challock, Ashford, Kent, UK). The plants were grown in pots in a heated greenhouse with supplementary lighting that maintained a 16 h light/ 8 h dark cycle with a minimum temperature of 20 °C. Plants were watered three times per week.

### 2.2. Elicitation of Gamay Cell Cultures and Gamay Leaves

Gamay cell suspensions that were 14-days-old from subculture were used for elicitation experiments. Cells were separated from their growth medium and washed by filtration under gentle vacuum in sterile conditions. Twenty grams of washed cells were transferred to a sterile 250 mL Erlenmeyer flask and re-suspended using 100 mL of Gamborg B5 medium supplemented with either 0.1 mM MJ, 50 mM of methyl-β-cyclodextrin (CD), or both together, as the elicitor. The cell suspensions were incubated for up to 96 h at 25 °C in darkness with orbital shaking at 110 rpm. After incubation, the cells were recovered by filtration as above, and stored at −80 °C until use for RNA and protein extraction. The spent medium was used immediately for stilbene analysis.

For experiments on the elicitation of whole leaves, three-weeks-old Gamay leaves from three independent plants were detached per treatment and time. Leaves of uniform size to fit in a Petri dish were selected from the same node position on each sampled stem. The petiole was quickly submerged in distilled water. The leaves were washed with abundant distilled water 4 times for 10 min, placed in Petri dishes with the adaxial face in contact with a sterile filter paper moistened with 3 mL of either 50 mM of CD or water, and incubated for up to 96 h at 25 °C in darkness. After incubation, three leaves from the same plant and treatment were pooled, then powdered with liquid N_2_ in a mortar and pestle and used for RNA and stilbene extraction. Thus, each biological replicate contained three leaves.

### 2.3. Stilbene Extraction and Quantitation by UHPLC-QqQ-MS/MS

Stilbenes from Gamay cell cultures accumulate mostly in the growth medium [12]. Thus, an aliquot of extracellular medium was diluted with 80% (*v*/*v*) methanol. For the leaf samples, stilbenes were extracted from 0.5 g of leaf tissue with 10 mL of pure methanol overnight on an orbital shaker at 25 °C in the dark. The samples were centrifuged at 8000× *g* and the recovered supernatant was evaporated in a speedVac system, resuspended in a small volume of 80% (*v*/*v*) methanol, and the insoluble material removed by bench-top centrifugation at 14,000× *g*.

Samples were filtered through a regenerated cellulose 0.2 µm Anopore syringe filter (Whatman, Little Chalfont, Buckinghamshire, UK) and analysed using an Agilent 1290 infinity UHPLC system coupled to an Agilent 6490 triple quadruple (QqQ) Mass spectrometer, through Jet Stream^®^ ion source (Agilent, Santa Clara, CA, USA), using the methodology described in [36]. The stilbenes were separated using a Zorbax Extended -C18 column (2.1 × 50 mm, 1.8 μm; 1200 bar maximal pressure). The optimized gradient conditions consisted in solvent A (0.05% trifluoroacetic acid) and solvent B (acetonitrile:methanol 70:30 with 0.05% trifluoroacetic acid) using the following gradient: 0 min 35% B, 2 min 35% B, 2.3 min 36.4% B, 3 min 37% B, 4 min 40% B, 5 min 65% B, and 6 min 65% B at constant flow rate of 0.4 mL·min^−1^.

Multiple reaction monitoring analysis mode was used for the quantitation of stilbenes by monitoring the precursor ions to the dominant product ions and using a multiple point calibration curve constructed with serial dilutions of stilbene standards.

### 2.4. Native Protein Extraction and PPC Assay

Five grams of elicited and control Gamay cells were homogenized using a potter Elvehjem with 10 mL extraction buffer (250 mM Sucrose, 50 mM HEPES, 5% glycerol, 10 mM ascorbic acid, 10 mM EDTA, 10 mM Sodium metabisulfite, 100 mM PMSF and Sigma protease inhibitor cocktail), pH 7.5, supplemented with 25 mM Sodium Fluoride, 1 mM Sodium Molydbate and 50 mM Sodium Pyrophosphate, to preserve the phosphorylation status of the proteins. The homogenate was centrifuged at 7700× *g* and the supernatant was ultracentrifuged at 110,000× *g* for 1 h at 4 °C. The freshly prepared soluble fraction containing native protein was used to determine PPC activity. Total protein concentration was measured with the RC DC protein assay (Bio-Rad, Madrid, Spain), based on the modified Lowry protein assay [37].

Native protein extracts from 48 to 72 h control and elicited Gamay cells were used for PPC kinetics assays. PPC activity was measured in a colorimetric assay with a coupled reaction, where oxaloacetic acid produced by carboxylation of phosphoenolpyruvate is converted to L-Malate by action of malate dehydrogenase (MDH) using NADH. The final reaction buffer consisted of 80 mM Tris-Sulfate pH 8.5, 10.5 mM Magnesium sulfate, 0.21 mM β-NADH, 10 mM sodium bicarbonate, 10 mM DTT, 6 U of porcine MDH and 0.6 µL of protein extract. The reaction was started by the addition of 1 mM of PEP. The decrease of A340 nm was measured using an Infinite M 200 microplate reader (TECAN, Männedorf, Switzerland), during 1 h at 25 °C. One unit of PPC activity is defined as a 1 µmol of NADH converted to 1 µmol of NAD^+^ in 1 min. Activity data were processed and converted to specific activity (U/mg protein) using the total amount of protein in the extract. The assay conditions were previously tested using a commercial microbial PPC. All reagents were obtained from Sigma Aldrich (San Luis, MO, USA). PPC activity was the average of six technical replicates performed using three biological replicates in separated assays.

### 2.5. RNA Extraction, cDNA Synthesis and RT-qPCR Analysis

Total RNA from Gamay cells was isolated as described elsewhere [38] from 1 g of elicited grapevine cells, and was quantified in a Nanodrop ND-1000 spectrophotometer (Thermo Scientific, Madrid Office, Madrid, Spain). Residual genomic DNA was removed by DNase I digestion with RNase-Free DNase I (Thermo Scientific). First-strand cDNA was synthesized from 1 µg of total RNA by a cDNA synthesis kit (RevertAid First Strand cDNA Synthesis Kit, Thermo Scientific) according to the manufacturer’s instructions.

Total RNA from Gamay leaves was isolated with Spectrum Plant Total RNA Kit (Sigma) from 100 mg of leaf powder. cDNA synthesis was performed with the Quantitect RT Kit (Qiagen Iberia, Barcelona, Spain) according to the manufacturer’s instructions.

The integrity of the RNA samples was checked by electrophoresis and the quality by spectrophotometry; only the samples showing no degradation with a 260/280 ratio between 1.9 and 2.1 were used.

SYBR Green Real-time PCR Master mixes (Thermo Scientific) were used for RT-qPCR in a StepOne plus instrument (Life Technologies). Gene-specific primers (Supplementary Materials, Table S1) for *VvPPCK1* (VIT_14s0036g00420.t01), *VvPPCK2* (VIT_05s0049g00950.t01), *VvPPC1* (VIT_12s0028g02180.t01), and *VvPPC2* (VIT_19s0014g01390.t01) were designed using Oligo Analyzer 3.1 (IDT, Integrated DNA technologies). Primers for *VvSTS29* (VIT_16s0100g01010) and *VvMYB14* (VIT_07s0005g03340) were as described in [33], *VvSTS36* (VIT_16s0100g01100) in [13] and *VvWRKY24* (VIT_08s0058g00690) in [34]. For each pair of primers, the reaction efficiency estimates were derived from a standard curve generated from a serial dilution of pooled cDNA. Transcript abundances were normalized with respect to the grapevine Elongation Factor-1 alpha (*VvEF-1α*) used as a reference gene, as described in [39,40]. We used three biological replicates per treatment, and the expression ratios calculated are displayed as the Log2 of ΔΔCt ratio between elicited and control at the given incubation time.

### 2.6. Determination of PPC Phosphorylation Change

To determine phosphorylation changes in PPC, three surrogate tryptic peptides, namely MASIDAQLR, LATPELEYGR, and LADLEAAPAAVAR, were selected based on the following criteria: all must be present in both isoforms 1 and 2, one (the first) must be susceptible of phosphorylation by PPCK according to the literature [14], whereas the others not thus serving as internal references. For an accurate quantitation, the selected peptides and their corresponding stable isotopically labelled (SIL) heavy versions (Arg U-13C6;U-15N4, providing 10 atomic mass unit (amu) increase) obtained from JPT Peptide Technologies (Berlin, Germany) as SpikeTidesTM L quality, were monitored by MRM using the transitions and collision energies indicated in the Supplementary Materials, Table S2, using an Agilent 1290 Infinity UHPLC system coupled to an Agilent 6490 triple quadrupole (QqQ) mass spectrometer through an Agilent Jet Stream^®^ ion source (Agilent, Santa Clara, CA, USA). Separation was carried out using an Agilent Zorbax Extend-C18 (2.1 × 50 mm, 1.8 μm particle size; 1200 bar maximal pressure; Agilent, Santa Clara, CA, USA). The mobile phase consisted in A: water with 0.1% formic acid; solvent B: 90% ACN, water with 0.1% formic acid. The flow rate was 0.4 mL per min throughout the following 7-min gradient: 0 min: 5% B, 3 min: 25% B, 6 min: 35% B, 6.5 min: 50% B, and 7 min: 90% B. The optimised ESI source parameters were: capillary voltage 3.5 kV, gas curtain temperature 150 °C, gas flow 15 L.min^−1^, cell acceleration voltage 4 V, and nebuliser (50 psi).

Protein extracts were resolved first by SDS-PAGE and the band corresponding to 110 kD was excised and in-gel trypsin digested as described previously [41]. The recovered peptide extract was spiked with 200 fmol of each SIL peptide, dried in a Speed-Vac centrifuge, resuspended in 10 µL of 0.1% formic acid and 5 µL were injected in the UHPLC-QqQ system. Chromatographic traces of each transition were analysed with Skyline v4.2 software (MacCoss Lab, University of Washington, Seatle, WA, USA) [42] to obtain the peak native/SIL peak area ratios.

### 2.7. Statistical Analysis

Test F was performed to determine homogeneity of variances. Significant differences for RT-qPCR data, MRM, and PPC enzyme activity assays were determined with the Student’s *t*-test.

## 3. Results

### 3.1. Grape VvPPCK Genes Are Down-Regulated in Response to Elicitation

Previous microarray-based transcriptome-wide analysis of the response to the MJ and CD elicitors in *Vitis vinifera* cv. Monastrell cell cultures, described in Almagro et al. (2014) [13], revealed the down-regulation of both of the *PPCK* genes in the grape genome, namely *VvPPCK1* (VIT_14s0036g00420.t01), *VvPPCK2* (VIT_05s0049g00950.t01). In order to validate these transcriptomic results, RNA from Gamay cell cultures treated with CD, MJ, and both together, was isolated and used to measure the transcript abundance of the genes of interest using RT-qPCR. We designed the gene specific primers to avoid conserved sequences shared between isogenes. Moreover, qPCR primers were also designed to quantify the transcript levels of the target substrates for PPCK, namely *VvPPC1* (VIT_12s0028g02180.t01), and *VvPPC2* (VIT_19s0014g01390.t01), which were also quantified for the same experimental conditions and samples.

**Figure 1:**
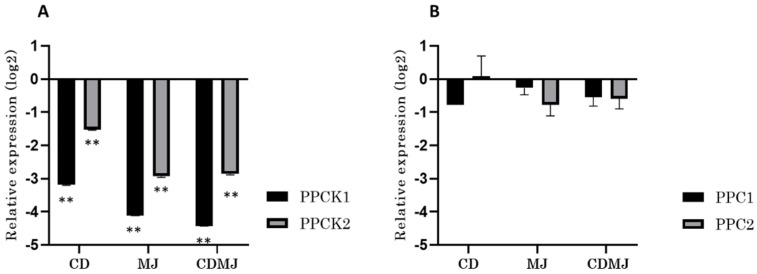
Analysis of grapevine *PPCK* and *PPC* genes expression in Gamay cell cultures in response to elicitation. (**A**) RT-qPCR analysis of *VvPPCK1* and *VvPPCK2* in cell cultures treated with elicitors CD, MJ, and CDMJ for 24 h. (**B**) RT-qPCR analysis of *VvPPC1* and *VvPPC2* in cell cultures treated with elicitors CD, MJ, and CDMJ for 24 h. Transcript levels were normalized to the reference gene *VvEF1-alpha*, and values are presented as relative expression to the control treatment. (* *p*-value < 0.05; ** *p*-value < 0.01).

**Figure 2:**
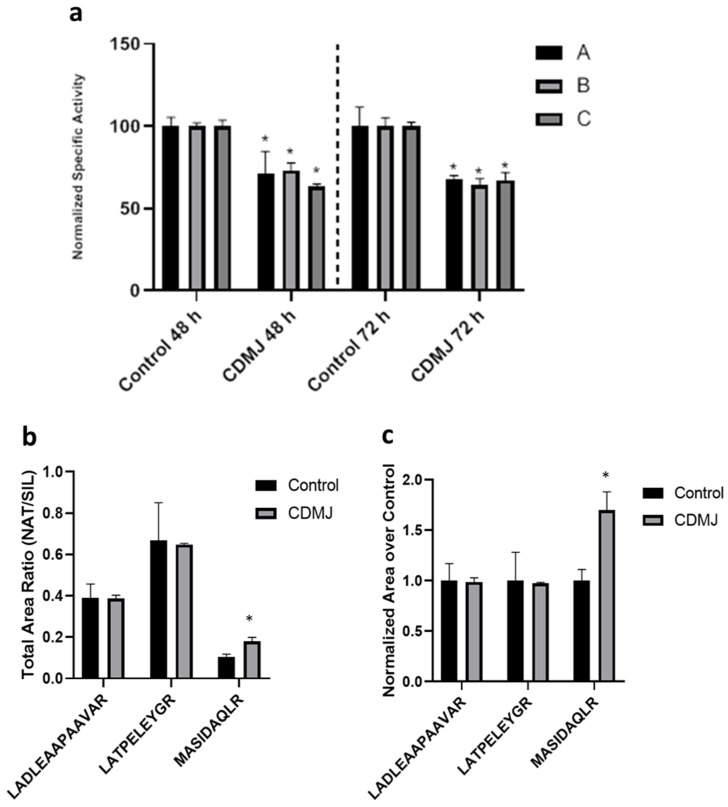
Determination of total extractable activity and changes in the phosphorylation levels of PPC in response to elicitation with the combined treatment CDMJ. (**a**) Specific activity (U/mg) of PPC in native extracts of grapevine Gamay cell cultures upon elicitation with CDMJ at 48 and 72 h. Each bar represents the result of one biological replicate (A, B, C) with triplicate assays normalized to its respective control. One biological replicate is an independent culture flask. (**b**,**c**) Relative abundance of PPC surrogate tryptic peptides in native extracts of grapevine Gamay cell cultures upon elicitation with CDMJ at 72 h. MASIDAQLR contains the conserved PPCK phosphorylation target Ser residue [14]. The two other peptides serve as internal reference. Peptides were recovered from an excised 110 kDa SDS-PAGE band digested in-gel with trypsin and spiked with a mixture of the SIL version of the targeted peptides. Each bar is the average of three biological replicates. (**b**): Abundance of each native peptide relative to its SIL version measured as the peak area ratio acquired in MRM mode. Differences between peptides can be due to the inaccuracy in quantification of the SIL peptides of “SpikeTidesTM L” quality. (**c**): To highlight the differences in the abundance of peptides between the control and CDMJ, data were normalized versus the control condition for each peptide. Significance of differences between control and CDMJ were determined using Student’s *t*-test (* *p* < 0.05).

*VvPPCK1* and *VvPPCK2* were repressed by between a 1.5- and 4.5-log2-fold change, in response to elicitation in each of the three treatments analysed (Figure 1A). By contrast, the levels of *VvPPC1* and *VvPPC2* remained almost unchanged in response to all three elicitation treatments (Figure 2A). The two *PPCK* genes responded to different extents to the various elicitation treatments applied. On the one hand, *VvPPCK1* and *VvPPCK2* responded strongly to MJ showing a −4.11 and −2.92 log2 fold-change, respectively (Figure 1A). Similar repression levels were observed for the combined CDMJ treatment for both genes when compared to the response to MJ alone (Figure 1A). Furthermore, *VvPPCK1* transcript levels were down-regulated more in response to elicitation relative to the control treatments than those of *VvPPCK2* (Figure 1A). In contrast, both *VvPPC* genes did not show significant changes (*p*-value > 0.05) in their transcript levels in response to the elicitation treatments, and the regulation of their transcript abundance between treatments varied only a little (Figure 1B).

### 3.2. Elicitation Leads to PPC Dephosphorylation and a Decrease in PPC Activity

To investigate if the reduction in *VvPPCK* transcripts may be linked to the redirection of PEP into the shikimate pathway to support increased stilbene synthesis mediated by changes in PPC, both enzyme activity and phosphorylation changes were measured in protein extracts from Gamay cell cultures elicited with CDMJ. We chose the CDMJ treatment to perform these determinations because the largest decrease in *VvPPCK* transcripts occurred in response to this treatment (Figure 1A), and stilbene production has previously been reported to be greatest in response to this elicitor combination [12,32,40]. PPC assays were performed at both 48 and 72 h after elicitation and phosphorylation changes at 72 h (Figure 2).

PPC activity decreased by between 25 and 35% (*p*-value < 0.05) in cell cultures treated with CDMJ. Abundance of the non-phosphorylated version MASIDAQLR increased by a 70% (*p*-value < 0.05) in the elicited cells while the abundance of the two unmuted internal reference peptides LATPELEYGR and LADLEAAPAAVAR (*p*-values = 0.96 and 0.84 respectively) did not significantly change with respect to control cells, thus dephosphorylated PPC increased by a 70%. PPC activity was similar at both elicitation time points tested, suggesting that the levels of PPC activity were not responding directly to the detected transcript level repression of the PPC regulatory enzyme VvPPCK at 24 h nor with the levels of PPC transcripts. This is consistent with the fact that PPCK participates in the PPC regulation via post-translational modification through phospho-/dephosphorylation.

### 3.3. PPCK Down-Regulation and Stilbene Accumulation also Occurs in Leaves in Response to CD Elicitation

The response to the CD elicitor in *V. vinifera* cv. Gamay and Monastrell berry cell cultures has been previously studied in relation to stilbene accumulation and the transcript level regulation of the associated biosynthetic genes [4,12,13,40,43]. However, the effect of this elicitor has never been tested against intact tissues or organs of grapevine plants. In order to determine whether the measured repression of *VvPPCK* genes promoted by elicitation (Figure 1A) was also reproducible in planta, or, by contrast, whether it is a unique characteristic of cell cultures, we designed and performed an elicitation assay using intact, detached Gamay leaves exposed to 50 mM of CD.

From each plant, three leaves were used per treatment and time, between 24 to 96 h of elicitation. Leaves from each treatment were pooled and used to determine the transcript levels of the *VvPPCKs* and *VvPPCs*, as well as the production of stilbenes.

**Figure 3:**
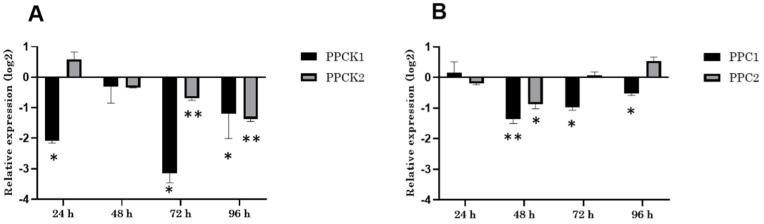
Analysis of grapevine *PPCK* and *PPC* genes expression intact Gamay leaf in response to CD elicitation. (**A**) RT-qPCR analysis of *VvPPCK1* and *VvPPCK2* and (**B**) RT-qPCR analysis of *VvPPC1* and *VvPPC2* in a leaf treated with CD, up to 96 h. Transcript levels were normalized to the reference gene *VvEF1-alpha* and are presented as values relative to the level of transcript quantified for the control treatment. (* *p*-value <0.05; ** *p*-value < 0.01).

Remarkably, the repression of the *VvPPCK1* and *VvPPC* transcripts at 24 h in the leaves was similar to the response detected in cell cultures (Figure 1), although not for *VvPPCK2*. While *VvPPCK1* was down-regulated after 24 h of elicitation treatment, *VvPPCK2* did not respond to 24 h of CD (Figure 3A). In addition, *VvPPC1* and *VvPPC2* levels did not change in response to CD after 24 h (Figure 3B), demonstrating that the CD elicitor treatment caused a very similar response for *VvPPCK1* and *VvPPC* genes measured in vitro using cell culture, and *in planta* using intact leaves.

At longer elicitation times, the transcript level of *VvPPCK1* in leaves followed an oscillatory pattern. Whereas after 24 h CD treatment *VvPPCK1* was down-regulated two-fold on a log2 scale, this repression was absent after 48 h of CD (Figure 3A). The lack of repression after 48 h was followed by further down-regulation after 72 h, with *VvPPCK1* repressed over three-folds on the log2 scale. Finally, after 96 h, the *VvPPCK1* level was once more very close to the level of the control treatment, although it was still down-regulated slightly relative to the control (Figure 3A). In contrast, the temporal response profile for *VvPPCK2* transcripts in leaves displayed a gradual and progressive decrease with the time of treatment, reaching its strongest down-regulation at 96 h, when the degree of repression of the transcript levels were close to those of *VvPPCK1*, although with much smaller error bars for *VvPPCK2* at 96 h relative to *VvPPCK1*.

Consistent with the response to CD measured for Gamay cell cultures (Figure 1), the *VvPPC* genes displayed either no or only a small and transient level of repression in response to elicitation treatment of leaves with CD (Figure 3B). The largest response for both *VvPPC1* and *VvPPC2* was detected after 48 h of CD treatment, when both transcripts were reduced by approximately one-fold (on the log2 scale) relative to the control treatment.

**Figure 4:**
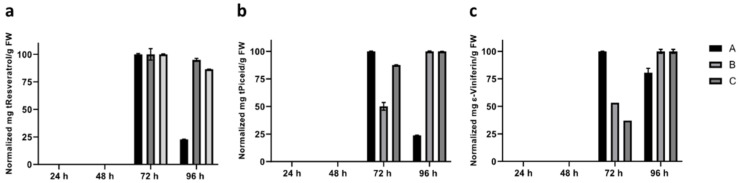
CD elicitation of intact Gamay leaves induced stilbene accumulation. Time-course of stilbenes accumulation in Gamay leaf treated with 50 mM of CD. The stilbenes tR (**a**), tPiceid (**b**) and ε-Viniferin (**c**) were detected in elicited leaves of three independent Gamay plants (A, B, and C). For a better comparison, the abundance of each compound was normalized to the highest value in each plant: % compound X in plant Y = mg/g FW of compound X in plant Y × 100/highest value of mg/g FW of compound X in plant Y. Error bars correspond to MRM analysis of three technical replicates.

Given that the induction of stilbene production in cell cultures occurred in concert with the repression of *VvPPCK* transcripts, stilbene production in the intact leaves in response to the CD treatment was determined (Figure 4). Stilbenes were accumulated in the CD treated intact Gamay leaves, and the accumulation was very similar pattern for the three independent plants measured (Figure 4). Stilbenes were undetectable by UHPLC-MS/MS after 24 h of CD treatment (Figure 4). The accumulation of resveratrol and piceid was increased after 48 h of treatment, and they reached their peak levels in the range of 10 and 20 µg/g fresh weight, respectively, after 72 h. By contrast, in the control treatment leaves, the stilbenes remain undetectable throughout the time-course experiment (Figure 4). In addition, accumulation of other stilbenes, including the bioactive trans-Resveratrol dimer, ε-Viniferin, was detected after 96 h of treatment (Figure 4).

### 3.4. Stilbene Production in Leaves Correlates with the Up-Regulation of VvSTS36 and VvSTS29

**Figure 5:**
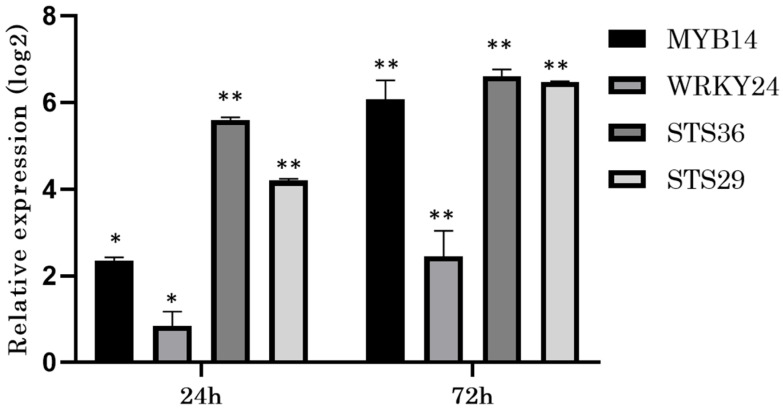
Analysis of expression of two grapevine *STS* paralogs and the TFs *VvMYB14* and *VvWRKY24* in Gamay cell cultures in response to CD elicitation. RT-qPCR analysis of *VvSTS36*, *VvSTS29*, *VvMYB14*, and *VvWRKY24* in Gamay cell cultures treated with 50 mM of CD at 24 and 72 h of incubation. Expression levels were normalized to *VvEF1-alpha*, and represented as relative expression to control treatment. (* *p*-value < 0.05; ** *p*-value < 0.01).

Previous studies in grapevine cell cultures analysed the regulation of the transcript abundances for several members of the STILBENE SYNTHASE (*VvSTS*) gene family in response to elicitation with CD, MJ, and CDMJ [13,32]. These studies reported the up-regulation of *VvSTS* transcript levels in response to elicitation, emphasizing especially *VvSTS36*, which was found to be highly responsive to CD treatment. On the other hand, *VvSTS29* was reported to be one of the most strongly induced members of the *VvSTS* gene family in grapevine leaves exposed to UV-radiation [33]. In order to further analyse the response of intact leaves to CD, we quantified the transcript levels for *VvSTS36* and *VvSTS39,* following treatment of leaves with CD for between 24 and 96 h (Figure 5). For comparison, we also measured the transcript level regulation of these *VvSTS* genes in the cell culture elicitation samples.

**Figure 6:**
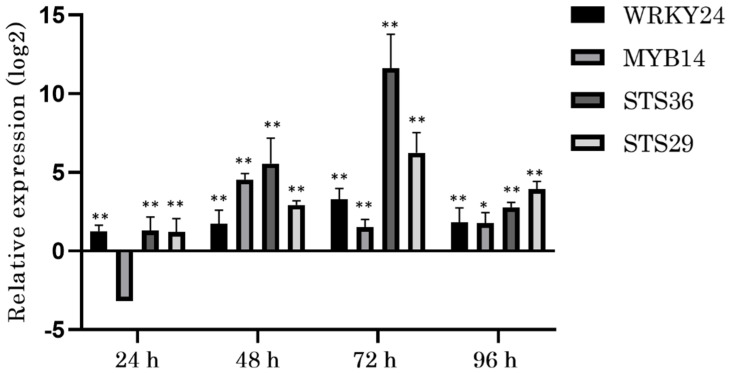
Analysis of expression two grapevine *STS* paralogs and the TFs *VvMYB14* and *VvWRKY24* intact Gamay leaf in response to CD elicitation. RT-qPCR analysis of *VvSTS36*, *VvSTS29*, *VvMYB14*, and *VvWRKY24* in Gamay leaves treated with 50 mM of CD up to 96 h. Expression levels were normalized to *VvEF1-alpha*, and represented as relative expression to control treatment. (* *p*-value < 0.05; ** *p*-value < 0.01).

*VvSTS* genes were induced more rapidly following elicitation in cell cultures as compared to the induction in intact leaves, which is consistent with the observed more rapid accumulation of stilbenes in cell cultures. After 24 h of CD treatment, both *VvSTS* genes were up-regulated by between four- and six-fold relative to the control in cell cultures, whereas in leaves these transcripts were not induced significantly after 24 h of elicitation (by comparing Figure 5 and Figure 6). However, after 72 h, the induction of *VvSTS* transcripts was greater in leaves than cell cultures for *VvSTS36*, whereas *VvSTS29* transcript levels responded to a similar extent in both tissues (by comparing Figure 5 and Figure 6). These results are consistent with those for stilbene accumulation, which started at 24 h for the cell cultures, but was not detected until after 72 h for the intact leaves, corresponding clearly with the timing of the induction of the *VvSTS* transcripts between the cell culture system and the intact leaves.

### 3.5. VvMYB14 and VvWRKY24 Transcription Factors Co-Express with VvSTS36 and VvSTS29 in Leaves and Cell Cultures in Response to CD Elicitation

Recently, a number of studies have identified transcription factors (TFs) involved in the regulation of the transcription of VvSTSs in response to different biotic and abiotic stresses [33,34,44]. The R2-R3 MYB-repeat family TF *VvMYB14*, and the WRKY-family TF *VvWRKY24* have been identified as positive regulators of the *VvSTS29* promoter using a dual luciferase reporter assay [23]. Here, we analysed the regulation of *VvMYB14* and *VvWRKY24* transcript levels in response to the CD elicitor treatment in both cell cultures and intact leaves. *VvMYB14* was rapidly up-regulated in cell cultures treated with CD (Figure 5). However, after 24 h of treatment, the fold-induction of *VvMYB14* was reduced relative to the control in the intact leaves (Figure 6). Meanwhile, *VvWRKY24* was induced marginally in both tissues after 24 h, without clear differences. This finding is consistent with previous results for *VvSTS* expression. After 24 h of CD treatment, the fold-induction relative to the control of *VvSTS36* and *VvSTS29* in leaves was much lower than the induction detected for the cell cultures, where both *VvSTS* genes were up-regulated between four- to five-folds on the log2 scale.

As the response of intact leaves to CD treatment was delayed relative to the response of the cell cultures, we explored the regulation of *VvMYB14*, *VvWRKY24, VvSTS36,* and *VvSTS29* transcript levels after longer incubation periods using the intact leaf system (by comparing Figure 5 and Figure 6). *VvMYB14* displayed its maximum peak of differential transcript accumulation relative to the control after 48 h of CD treatment (Figure 6). This *VvMYB14* peak preceded the maximal induction of both *VvSTS* transcripts after 72 h, consistent with the known role of *VvMYB14* in positively activating the *VvSTS29* promoter (Figure 6). This timing was also coincident with the highest levels of accumulation of the stilbenes after 72 h for the intact leaves (Figure 4). In contrast to *VvMYB14* transcripts peaking ahead of *VvSTS* levels by 24 h, the peak fold-change of *VvWRKY24* transcripts relative to the control matched the timing of the highest *VvSTS* fold-induction after 72 h. However, the fold-change for *VvWRKY24* after 72 h of CD treatment was lower than that of *VvMYB14* after 48 h (Figure 6). The fold-induction of both TFs relative to the control decreased after 96 h, and this reduced fold-induction was also mirrored for the *VvSTS* genes (Figure 6).

## 4. Discussion

### 4.1. The Repression of Grapevine PPCK in Response to Elicitors May Reduce PEP Flux through PPC and Promote Flux into the Shikimate Pathway to Support Stilbene Biosynthesis

The application of different types of stress to grapevine cell cultures and plants results in the induction of *VvSTS* genes and the accumulation of stilbene phytoalexins [13,32,45,46,47]. Elicitation experiments using grapevine cell cultures have elucidated the transcriptome-wide responses that promote stilbene synthesis, which includes the up-regulation of genes encoding enzymes of the shikimate and phenylpropanoid pathways, in addition to induction of *VvSTS* genes [13]. Nonetheless, the published microarray data revealed additional differentially expressed genes encoding enzymes outside these pathways, whose role in this response had not been identified previously. In this context, the repression of the *VvPPCK* genes in response to elicitor treatment revealed a previously unanticipated role for this regulatory protein kinase in controlling the diversion of primary metabolism towards stilbene biosynthesis.

PPCK is the protein kinase that phosphorylates PPC and adjust the allosteric properties of PPC, making it less sensitive to feedback inhibition by L-malate, and thus more active in vivo in the face of rising malate concentrations due to its own activity. To date, PPCK has mainly been studied in relation to its function as a key regulator of PPC in the CAM and C_4_ photosynthetic adaptation of primary CO_2_ fixation [20,48]. In particular, the circadian clock control of PPCK de novo transcription and translation each night in the CAM plant *Kalanchoë fedtschenkoi* has been demonstrated to be crucial for optimized and sustained PPC activity throughout the dark period during primary atmospheric CO_2_ fixation for CAM [21]. In addition, PPCK and its target protein PPC were found to be induced in response to both nutrient deprivation and/or toxic metal stress in the model C_3_ species *Arabidopsis thaliana* [29]. The response of PPCK and PPC to these abiotic stress treatments was hypothesized to promote the reprogramming of primary metabolism to support biosynthetic requirements and/or the accumulation and excretion of organic acids from roots for soil acidification and chelation of cations [29]. Here, we provide evidence that the down-regulation of *VvPPCK* genes in grapevine cell cultures and intact leaves in response to elicitation occurs in concert with the accumulation of stilbenoids. The most logical explanation for the repression of *VvPPCK* is that it drives improved PEP availability, thereby promoting the flux of metabolism into the shikimate pathway, which in turn provisions the early precursor molecules for all polyphenol biosynthesis.

The elicitors CD and MJ, either applied separately or in combination, promoted a significant reduction in the transcript levels of both *VvPPCK* genes, but not the *PPC*s, after 24 h of treatment of the cell culture (Figure 1). This response was also coincident with a marked reduction in the total extractable activity of PPC and increase in dephosphorylated PPC (Figure 2), although it should be noted that alterations in *PPCK* transcript levels have not previously been shown to lead to reduced total extractable activity of PPC. These results do however lead to the hypothesis that the reduction in *VvPPCK* transcripts and PPC activity would support the redirection of PEP flux towards other metabolic destinations, such as shikimate pathway. Moreover, according to the existing microarray data, elicitation promoted the up-regulation of transcripts encoding enolase (VIT_17s0000g04540), the glycolytic enzyme that synthesizes PEP, and transcripts for several pentose phosphate pathway enzymes [13]. Assuming that the detected transcript level changes resulted in coordinated alterations in the activity of the encoded enzymes, these changes are predicted to promote the synthesis of PEP and production of D-erythrose 4-phosphate, which are the two initial substrates required for flux into the shikimate pathway.

Altogether, the published results and our current data support the proposal that elicitation with CD, MJ, or both, results in widespread metabolic reprogramming that leads to the diversion of metabolic flux from sugars in primary metabolism towards the biosynthesis of stilbenoids. In grapevine cells, the amount of resveratrol produced in response to elicitation by CD alone, or combined with MJ (ca. 2–4 g/L, accounting for 1.5–2.9 g carbon/L [40]), may represent between 17.5 and 35% of conversion, respectively, of the sucrose supplied to the cell culture (20 g/L, accounting for 8.4 g carbon/L). Thus, our findings for *VvPPCK* repression and the coincident reduction in PPC activity and increase in PPC dephosphorylation in response to elicitor treatment, and in coordination with increased stilbene accumulation, support the conclusion that VvPPCK and PPC represent a key point in the metabolic crossroads between primary and secondary metabolism.

The response of the grapevine cell cultures to elicitor treatment was found to be largely reproducible in planta using grapevine intact leaves. Specifically, the elicitation of leaves with CD also led to the down-regulation of *VvPPCK* after only 24 h of treatment (Figure 3). Transcript levels for the *VvPPCK1* isogene displayed an oscillatory pattern of differential abundance relative to the control treatment over time, with the most dramatic repression detected at 24 and 72 h (Figure 3). In contrast to the results for the cell cultures, *VvPPCK2* transcript levels were unchanged relative to the control after 24 h in leaves, but *VvPPCK2* did undergo a gradual decrease in transcript abundance over time, reaching its maximal down-regulation after 96 h (Figure 3). *PPCK* transcripts in CAM, C_4,_ and C_3_ species are differentially abundant over the light/dark cycle, with the CAM-specific *PPCK* gene being under robust circadian clock control [20,21,49,50]. *V. vinifera* is a C_3_ plant in which the *PPCK* genes might be predicted to be used specifically in different tissue or organs, and/or in response to different environmental and internal signals. In this work, our experimental conditions used darkness for the cell cultures in order to ensure that there were no light/dark-dependent changes in *PPCK*. Although comparison between the cell culture and leaf experiments shows an overall similar response to elicitor treatment in terms of the transcript levels of the *VvPPCK* and *VvPPC* genes, there were however also subtle differences in the temporal profiles of the responses of these genes. In the cell culture system, responses were rapid, with large fold-changes relative to the control already evident after 24 h, and sustained with time. In contrast, in the intact leaf experiments, the responses varied with the duration of the exposure to the elicitor treatment. In the cell culture system, the strong repression within 24 h of both *VvPPCKs*, but not the *VvPPCs*, may be sufficient to mediate the increased flux of PEP into the shikimate pathway for stilbene synthesis, whereas in the intact leaves, the slight down regulation observed for *VvPPC* in addition to the repression of the *VvPPCKs*, may also contribute to the change in metabolic flux of PEP towards stilbenes, especially after 48 h when the level of the *VvPPCKs* was similar to the control.

### 4.2. Stilbene Accumulation in Response to CD

We applied our CD elicitation strategy to Gamay intact leaves as a representative model for the in planta response of grapevine to this elicitor. These findings provide a valuable contrast to the cell culture system, which is an in vitro cell suspension culture derived originally from callus generated using grape berry cells. Previous studies have reported stilbene accumulation in grapevine leaves and berries exposed to UV-radiation [51,52], mechanical damage [2], or the application of chemical treatments, such as aluminium chloride [53].

Strikingly, Gamay leaves responded to CD elicitation through not only synthesizing tR, but also by producing several other bioactive stilbenes (Figure 4). After 72 h of treatment with CD, trans-Piceid and tR were the first stilbenes to accumulate in the Gamay leaves, followed by a decrease of tR accumulation that was followed by Ɛ-Viniferin accumulation at 96 h (Figure 4). This pattern of accumulation of tR and its derivatives followed a similar temporal pattern to that observed previously for Gamay cell cultures, which showed a maximal extracellular accumulation in the growth medium at 72 h, which was also detected after 96 h of incubation [32]. In the intact leaves measured here, the resveratrol amount decreased relative to 72 h at the 96 h timepoint, most likely as a consequence of it being converted to its dimer Ɛ-Viniferin. Interestingly, it has previously been observed that the glycosylated forms, trans-piceid and cis-piceid, were produced constitutively in Gamay cell cultures and not affected significantly by CD treatment [12]. This constitutive accumulation was not detected in Gamay leaves, suggesting that the leaves initiated the de novo biosynthesis of stilbenes in response to CD. No other stilbenes found in grapevine and targeted with our analysis method, such as piceatannol and pterostilbene, were detected.

It is well documented that when grapevines are subjected to biotic or abiotic stresses, resveratrol is quickly synthetized but its accumulation is transient [11,45,54,55,56,57,58]. Those findings are further supported by the accumulation profile observed here for CD elicited leaves. In contrast with other kinds of abiotic or biotic stresses, CD induces a constant and progressive accumulation of trans-resveratrol in cell cultures [4,12,32,40]. This effect could be promoted by the sequestering of tR in CD ring, which acts as an adsorbent agent, reducing the effective resveratrol concentration.

Our results demonstrate the ability of CD to elicit the progressive synthesis and accumulation of stilbenes, not only in Gamay cell cultures, but also in Gamay leaves. These current findings therefore confirm that Gamay cell cultures are a representative model for in planta cell signalling responses to stress.

### 4.3. VvSTS36 and VvSTS29 Are Gradually Induced in Response to CD

To build on our discovery that the elicitation of both cell cultures and leaves with CD led to the repression of *VvPPCK* genes in concert with stilbene accumulation, we analysed the regulation of *VvSTS* genes in both tissues. Thus, we were able to examine whether the induction of key genes associated with stilbene synthesis and accumulation occurred in a coordinated manner with the measured changes in *VvPPCK*, which also occurred in concert with a change in the primary metabolism flux towards the shikimate pathway by improving the availability of PEP from glycolysis.

Stilbene synthases are encoded by a large gene family in grapevine, classified in three different sub-families, A, B, and C, from which group B members are highly responsive to both wounding and UV-C radiation abiotic stresses in grapevine leaves [47]. Within this classification, both *VvSTS29* and *VvSTS36* belong to group B. Microarray analysis studies have shown strong induction of *VvSTS36* and other STSs in a grapevine cell culture system elicited with CD [13]. In addition, Höll et al. (2013) [33] reported rapid and strong induction after 24 h of *VvSTS29* in leaves exposed to UV-C and downy mildew infection.

The transcript level regulation of *VvSTS29* and *VvSTS36* in Gamay cell cultures upon CD treatment revealed strong induction only after 24 h of elicitor treatment. This was followed by a further moderate increase between 24 and 72 h (Figure 5). However, in Gamay leaves, the transcripts levels of both *VvSTS* genes displayed low levels of induction at 24 h after CD treatment, which gradually increased to a peak after 72 h (Figure 6). This *VvSTS* transcript level response to CD also supports the hypothesis that the leaves display an accumulative response over time, which contrasts with the previously reported response of leaves to UV-C stress, where *VvSTS29* responded maximally after 24 h followed by a rapid decrease by 48 h [33]. Furthermore, the elicitation response of *VvSTS36* in leaves was double that of *VvSTS29*, supporting the conclusion that the CD stimulus promoted *VvSTS36* induction much more strongly than it did *VvSTS29* in leaves.

### 4.4. VvMYB14 and VvWRKY24 Induction Precedes That of VvSTS and the Timing of Peak Stilbene Accumulation

Recently, the grapevine TFs *VvMYB14*, *VvMYB1,5* and *VvWRKY24* have been reported to function in the regulation of *VvSTS* genes in response to stress in grapevine leaves [33,34]. Microarray analysis of the transcriptome [13] also supported the possibility of an important role for *VvMYB14* in the response to CD in grapevine cell cultures. Here we found that *VvMYB14* was up-regulated after 24 h following CD treatment of cell cultures, and was co-ordinately accumulated along with transcripts for *VvSTS36* and *VvSTS29* (Figure 5). In contrast, the response of *VvWRKY24* to CD after 24 h did not correspond as clearly with the initial and strong induction of *VvSTS29* and *VvSTS36* after 24 h.

*VvMYB14* levels in CD-treated leaves appeared to be repressed slightly at 24 h, and then underwent a very strong induction between 24 and 48 h, going from negative fold-induction to 23-fold induction relative to the control treatment after 48 h (Figure 6). This dramatic fold-induction of *VvMYB14* at 48 h preceded the increase in both *VvSTS* transcripts (Figure 6) and the peak of resveratrol accumulation (Figure 4). *VvMYB14* transcript levels decreased after 72 h, but, at that time, *VvWRKY24* levels reached their maximum (Figure 6). Vanozzi et al. (2018) [34] demonstrated that *VvMYB14* and *VvWRKY24* were both singular positive regulators of *VvSTS29* using luciferase as reporter gene under the control of the *pSTS29* promoter region. Transient expression of these TFs in cell culture, either singly or combined, resulted in similar levels of luciferase activity. If we therefore assume that *VvSTS29* expression responds to the level of the most strongly expressed of these TFs, either *VvMYB14* or *VvWRKY24*, then the transcript abundance profile of *VvSTS29* in response to CD elicitation in leaves can be explained by the abundance profiles of these TFs. Likewise, the transcript abundance profile of *VvSTS36* also corresponded well with the profile of these two TFs. These findings point to the possibility of redundancy within the mechanism of regulation of at least these two *VvSTS* paralogs, ensuring high levels of *VvSTS* up-regulation and production of stilbenes in response to stress.

Stresses such as wounding, UV-C irradiation, or a fungal infection of the leaf disks have been shown to cause a progressive accumulation of *VvMYB14* in advance of the *VvSTS* peak [33]. Similarly, we have found that *VvMYB14* accumulated ahead of *VvSTS* induction and stilbene accumulation in response to CD elicitor both in cell cultures and whole leaf. In addition, Jiang et al. 2019 [44] described a novel mechanism for the negative regulation of the *VvSTS15/21* promoter when resveratrol accumulation reached a threshold level through interaction between *VvMYB14* and the negative regulator *VvWRKY8*. However, this response was unlikely to be occurring in the experiments described here in response to CD elicitation, as CD can sequester resveratrol, and thereby promotes the characteristic response of stilbene accumulation.

## 5. Conclusions

All studies performed to date on the regulation of stilbene accumulation in grapevine in response to elicitor treatments and other stressors have focused on the specific branch point of the conversion of the phenylpropanoid precursor p-coumaroyl CoA or cinnamoyl CoA the corresponding stilbene, including the regulation of *VvSTS* genes and the coordinated regulation of the *VvMYB* and *VvWRKY* TFs that modulate their expression. Assuming STS as the unique (up)regulated metabolic step to induce synthesis of stilbenes, a strong competition for the phenylpropanoid precursors between several other metabolic pathways would occur and thus become negatively affected. An increase of the metabolic flux into and through the shikimate and phenylpropanoid pathways would be a powerful mechanism to avoid or minimize this competition. Notably, key steps within both pathways were shown to be up-regulated at the level of their transcripts by Almagro et al. (2014) [13]. However, an increased capacity for flux through both pathways would most likely have no positive effect downstream towards stilbenes unless the availability of the early precursors PEP and erythrose 4-phosphate was increased. Thus, PEP is likely to represent a crucial metabolic crossroad between primary or secondary metabolism, whereby the onwards metabolism of PEP towards its possible metabolic destinations responds both to external stimuli and cellular requirements. Our work revealed a novel role for the PPC regulatory protein kinase PPCK in the redirection of primary metabolism towards stilbenes in response to elicitor treatment in grapevine. Furthermore, we measured this response of *VvPPCK* both using in vitro cell cultures and in planta for intact leaves. These findings support the hypothesis that* VvPPCK* plays a pivotal regulatory role in the control metabolic flux of the central metabolite PEP between primary and secondary metabolism towards stilbene biosynthesis in response to stress.

## Supplementary Materials

The following are available online at https://www.mdpi.com/article/10.3390/biom11111641/s1, Table S1: List of primers used in this study. Table S2: Transition list used in MRM experiments.
