# Supplementary material for: Down-Regulation of Phosphoenolpyruvate Carboxylase Kinase in Grapevine Cell Cultures and Leaves Is Linked to Enhanced Resveratrol Biosynthesis
Source: Biomolecules. 2021 Nov 5;11(11):1641. doi: 10.3390/biom11111641 (PMC8615455; doi:10.3390/biom11111641)
Supplement: Supplementary file 1 [file biomolecules-11-01641-s001.zip › biomolecules-1404121-supplementary.pdf]

Table S1. Primers for qRT-PCR expression analysis in *V.vinifera* Gamay leaves and cell cultures

Name	ID	Sequence (5'-3')
VvMYB14 Fw	VIT_07s0005g03340	TCTGAGGCCGGATATCAAAC
VvMYB14 Rev		GGGACGCATCAAGAGAGTGT
VvWRKY24 Fw	VIT_08s0058g00690	ATTCAAGCACTAGTATGAACAGAGCAG
VvWRKY24 Rev		CCTTGTTGCCTTGGCATGA
VvSTS36 Fw	VIT_16s0100g01100	CGCCAGGAGATAATCACTGCT
VvSTS36 Rv		GCACCAGGCATTTCTACACC
VvSTS29Fw	VIT_16s0100g01010	GGTTTTGGACCAGGCTTGACT
VvSTS29Fw		GAGATAAATACCTTACTCCTATTCAAC
VvPPCK1Fw	VIT_14s0036g00420.t01	GCCGGAGTGATTTTGTACGTA
VvPPCK1Rv		GATTCTCGTCGGAAACCTCAA
VvPPCK2Fw	VIT_05s0049g00950.t01	TACATAATGCTGGCCGGTATC
VvPPCK2Rv		CTTTGCTGCCGTTGATACCGA
VvPEPC1Fw	VIT_12s0028g02180.t01	GCGGCAAGTTTCTACCTTTGGAC
VVPEPC1Rv		CTGCGTTCCTCAGACCATTCTCG
VvPEPC2Fw	VIT_19s0014g01390.t01	GGAGGACTTGGAAATGTGTTGACG
VvPEPC2Rv		CAGCAAAATCACCCTTCTTCAACT
VvEF1-alphaFw		GAACTGGGTGCTTGATAGGC
VvEF1-alphaRv		AACCAAAATATCCGGAGTAAAAGA

Table S2. Parameters for MS intensity acquisition in MRM mode in an Agilent QqQ instrument.

Protein	Peptide sequence	Charge	Precursor Ion (m/z)	Product Ion (m/z)	Fragment ion	Dwell	Fragmentor	Collision Energy (eV)	Cell Accelerator Voltage	Polarity
PEPC	LADLEAAPAAVAR.heavy	2+	639.358011	865.476561	y9	10	380	20.7	4	Positive
PEPC	LADLEAAPAAVAR.heavy	2+	639.358011	736.433968	y8	10	380	20.7	4	Positive
PEPC	LADLEAAPAAVAR.heavy	2+	639.358011	684.356282	b7	10	380	20.7	4	Positive
PEPC	LADLEAAPAAVAR.heavy	2+	639.358011	665.396854	y7	10	380	20.7	4	Positive
PEPC	LADLEAAPAAVAR.heavy	2+	639.358011	594.35974	y6	10	380	20.7	4	Positive
PEPC	LADLEAAPAAVAR.light	2+	634.353876	855.468292	y9	10	380	20.7	4	Positive
PEPC	LADLEAAPAAVAR.light	2+	634.353876	726.425699	y8	10	380	20.7	4	Positive
PEPC	LADLEAAPAAVAR.light	2+	634.353876	684.356282	b7	10	380	20.7	4	Positive
PEPC	LADLEAAPAAVAR.light	2+	634.353876	655.388585	y7	10	380	20.7	4	Positive
PEPC	LADLEAAPAAVAR.light	2+	634.353876	584.351471	y6	10	380	20.7	4	Positive
PEPC	LATPELEYGR.heavy	2+	579.80508	974.482939	b9	10	380	18.8	4	Positive
PEPC	LATPELEYGR.heavy	2+	579.80508	974.481706	y8	10	380	18.8	4	Positive
PEPC	LATPELEYGR.heavy	2+	579.80508	873.434027	y7	10	380	18.8	4	Positive
PEPC	LATPELEYGR.heavy	2+	579.80508	776.381263	y6	10	380	18.8	4	Positive
PEPC	LATPELEYGR.heavy	2+	579.80508	286.176132	b3	10	380	18.8	4	Positive
PEPC	LATPELEYGR.light	2+	574.800945	964.482939	b9	10	380	18.8	4	Positive
PEPC	LATPELEYGR.light	2+	574.800945	964.473437	y8	10	380	18.8	4	Positive
PEPC	LATPELEYGR.light	2+	574.800945	863.425758	y7	10	380	18.8	4	Positive
PEPC	LATPELEYGR.light	2+	574.800945	766.372994	y6	10	380	18.8	4	Positive
PEPC	LATPELEYGR.light	2+	574.800945	286.176132	b3	10	380	18.8	4	Positive
PEPC	MASIDAQLR.heavy	2+	507.767443	812.450012	y7	10	380	16.6	4	Positive
PEPC	MASIDAQLR.heavy	2+	507.767443	725.417983	y6	10	380	16.6	4	Positive
PEPC	MASIDAQLR.heavy	2+	507.767443	612.333919	y5	10	380	16.6	4	Positive
PEPC	MASIDAQLR.light	2+	502.763309	802.441743	y7	10	380	16.6	4	Positive
PEPC	MASIDAQLR.light	2+	502.763309	715.409714	y6	10	380	16.6	4	Positive
PEPC	MASIDAQLR.light	2+	502.763309	602.32565	y5	10	380	16.6	4	Positive
